# Towards a guideline for evaluation metrics in medical image segmentation

**DOI:** 10.1186/s13104-022-06096-y

**Published:** 2022-06-20

**Authors:** Dominik Müller, Iñaki Soto-Rey, Frank Kramer

**Affiliations:** 1 IT-Infrastructure for Translational Medical Research, University of Augsburg, Augsburg, Germany; 2 Medical Data Integration Center, Institute for Digital Medicine, University Hospital Augsburg, Augsburg, Germany

**Keywords:** Biomedical image segmentation; Semantic segmentation; Medical Image Analysis, Reproducibility, Evaluation, Guideline, Performance assessment

## Abstract

In the last decade, research on artificial intelligence has seen rapid growth with deep learning models, especially in the field of medical image segmentation. Various studies demonstrated that these models have powerful prediction capabilities and achieved similar results as clinicians. However, recent studies revealed that the evaluation in image segmentation studies lacks reliable model performance assessment and showed statistical bias by incorrect metric implementation or usage. Thus, this work provides an overview and interpretation guide on the following metrics for medical image segmentation evaluation in binary as well as multi-class problems: Dice similarity coefficient, Jaccard, Sensitivity, Specificity, Rand index, ROC curves, Cohen’s Kappa, and Hausdorff distance. Furthermore, common issues like class imbalance and statistical as well as interpretation biases in evaluation are discussed. As a summary, we propose a guideline for standardized medical image segmentation evaluation to improve evaluation quality, reproducibility, and comparability in the research field.

## Introduction

In the last decade, research on artificial intelligence has seen rapid growth with deep learning models, by which various computer vision tasks got successfully automated through accurate neural network classifiers [1]. Evaluation procedures or quality of model performance are highly distinctive in computer vision between different research fields and applications.

The subfield medical image segmentation (MIS) covers the automated identification and annotation of medical regions of interest (ROI) like organs or medical abnormalities (e.g. cancer or lesions) [2]. Various novel studies demonstrated that MIS models based on deep learning revealed powerful prediction capabilities and achieved similar results as radiologists regarding performance [1, 2]. Clinicians, especially from radiology and pathology, strive to integrate deep learning based MIS methods as clinical decision support (CDS) systems in their clinical routine to aid in diagnosis, treatment, risk assessment, and reduction of time-consuming inspection processes [1, 2]. Throughout their direct impact on diagnosis and treatment decisions, correct and robust evaluation of MIS algorithms is crucial.

However, in the past years a strong trend of highlighting or cherry-picking improper metrics to show particularly high scores close to 100% was revealed in scientific publishing of MIS studies [3–7]. Studies showed that statistical bias in evaluation is caused by issues reaching from incorrect metric implementation or usage to missing hold-out set sampling for reliable validation [3–11]. This led to the current situation that various clinical research teams are reporting issues on model usability outside of research environments [4, 7, 12–16]. The use of faulty metrics and missing evaluation standards in the scientific community for the assessment of model performance on health-sensitive procedures is a large threat to the quality and reliability of CDS systems.

In this work, we want to provide an overview of appropriate metrics, discuss interpretation biases, and propose a guideline for properly evaluating medical image segmentation performance in order to increase research reliability and reproducibility in the field of medical image segmentation.

## Main text

### Evaluation metrics

Evaluation of semantic segmentation can be quite complex because it is required to measure classification accuracy as well as localization correctness. The aim is to score the similarity between the predicted (prediction) and annotated segmentation (ground truth). Over the last 30 years, a large variety of evaluation metrics can be found in the MIS literature [10]. However, only a handful of scores have proven to be appropriate and are used in a standardized way [10]. This work demonstrates and discusses the behavior of the following common metrics for evaluation in MIS:

- F-measure based metrics like Dice Similarity Coefficient (DSC) and Intersection-over-Union (IoU)
- Sensitivity (Sens) and Specificity (Spec)
- Accuracy / Rand Index (Acc)
- Receiver Operating Characteristic (ROC) and the area under the ROC curve (AUC)
- Cohen’s Kappa (Kap)
- Average Hausdorff Distance (AHD)

**Fig. 1:**
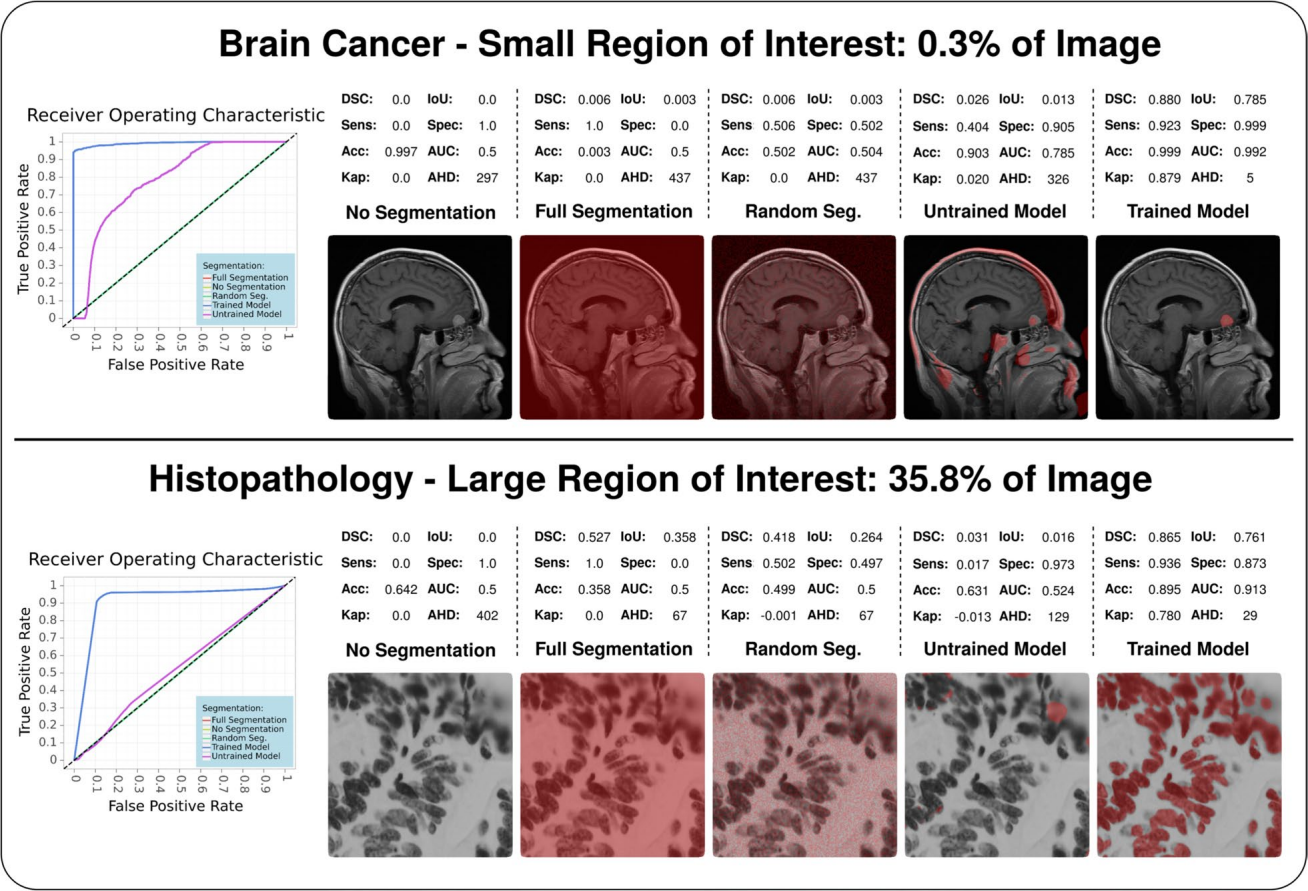
Demonstration of metric behavior in the context of different-sized ROIs compared to the total image. The figure is showing the perks of F-measure based metrics like DSC as well as IoU and the inferiority of Rand index usage. Furthermore, the small ROI segmentation points out that metrics like accuracy have no value for interpretation in these scenarios, whereas the large ROI segmentation indicates that small percentage variance can lead to a risk of missing whole instances of ROIs. The analysis was performed in the following scenarios and common MIS use cases. Scenarios: No segmentation (no pixel is annotated as ROI), full segmentation (all pixels are annotated as ROI), random segmentation (full random-based annotation), untrained (after 1 epoch during training) and trained model (fully fitted model). Use cases: Small ROIs via brain tumor detection in magnetic resonance imaging and large ROIs via cell nuclei detection in pathology microscopy

**Fig. 2:**
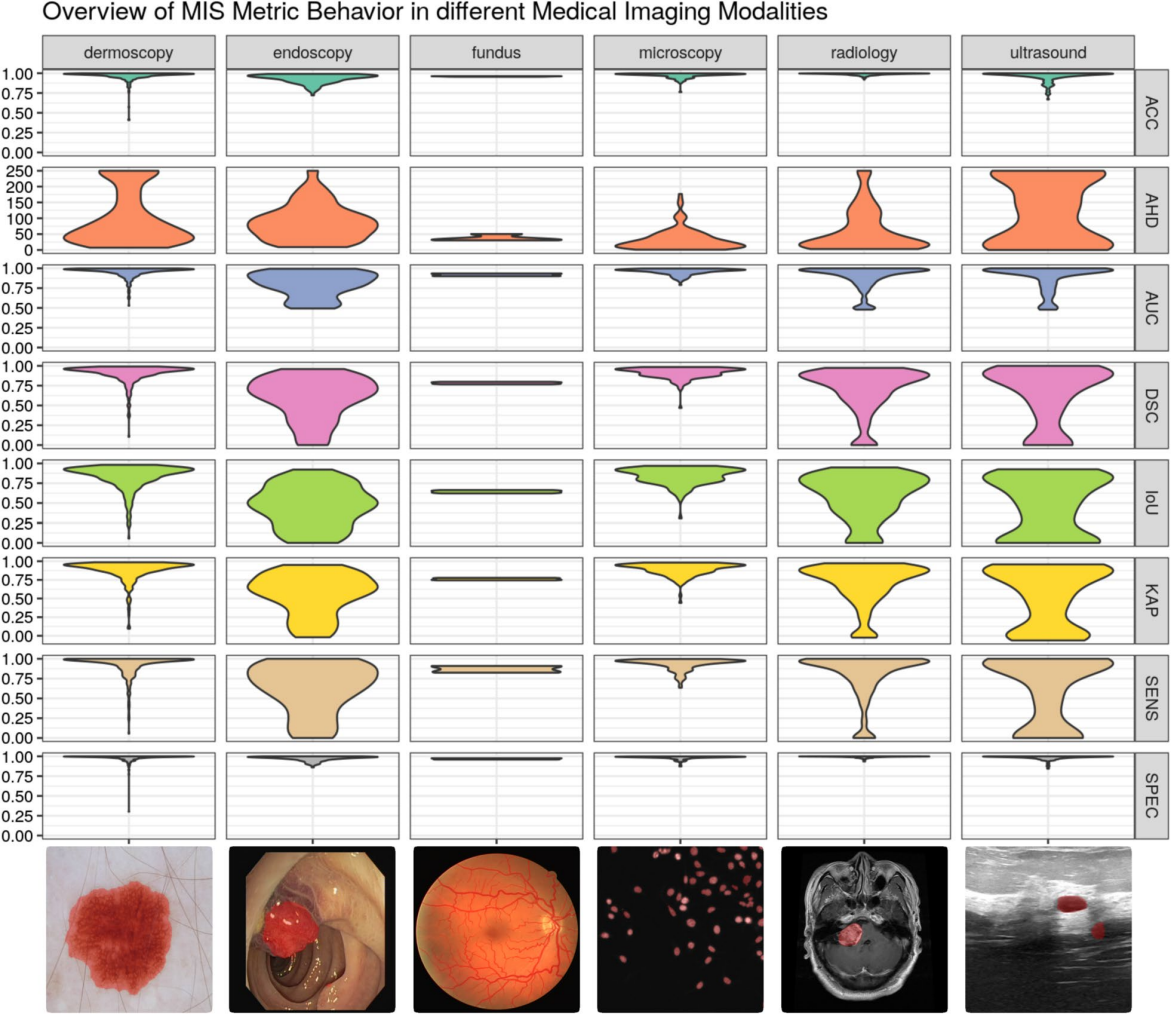
Demonstration of metric behavior for a trained segmentation model in the context of different medical imaging modalities. The figure is showing the differences between metrics based on distance like AHD, with true negatives like Accuracy, and without true negatives like DSC. Each subplot illustrates a violin plot which visualizes the resulting scoring distribution of all testing samples for the corresponding metric and modality. For visualization purposes, AHD was clipped to a maximum of 250 (affected number of samples per dataset: dermoscopy 2.0%, endoscopy 0.3%, fundus 0.0%, microscopy 0.0%, radiology 0.5%, and ultrasound 2.5%)

In detail descriptions of these metrics are presented in the Appendix. The behavior of the metrics in this work is illustrated in Fig. 1 and Fig. 2 which demonstrate the metric application in multiple use cases.

### Class imbalance in medical image segmentation

Medical images are infamous in the field of image segmentation due to their extensive class imbalance [10, 17]. Usually, an image in medicine contains a single ROI taking only a small percentage of pixels in the image, whereas the remaining image is all annotated as background. From a technical perspective for machine learning, this scenario entails that the model classifier must be trained on data composed of a very rare ROI class and a background class with often more than 90% or even close to 100% prevalence. This extreme inequality in class distribution affects all aspects of a computer vision pipeline for MIS, starting from the preprocessing, to the model architecture and training strategy up to the performance evaluation [18].

In MIS evaluation, class imbalance significantly affects metrics which include correct background classification. For metrics based on the confusion matrix, these cases are defined as true negatives. In a common medical image with a class distribution of 9:1 between background and ROI, the possible number of correct classifications is extensively higher for the background class compared to the ROI. Using a metric with equal true positive and true negative weighting results in a high-ranking scoring even if any pixel at all is classified as ROI and, thus, significantly biases the interpretation value. This behavior can be seen in metrics like Accuracy or Specificity which present always significantly high scorings in any MIS context. Therefore, these metrics should be avoided for any interpretation of segmentation performance. Metrics that focus on only true positive classification without a true negative inclusion provide better performance representation in a medical context. This is why the DSC and IoU are highly popular and recommended in the field of MIS.

### Influence of the region-of-interest size on evaluation

The size of an ROI and the resulting class imbalance ratio in an image demonstrates an anti-correlation to evaluation complexity for interpretation robustness. In the medical context, the ROI size is determined by the type in terms of the medical condition and the imaging modality. Various types of ROIs can be relevant to segment for clinicians. Whereas organ segmentation, cell detection, or a brain atlas take up a larger fraction of the image and, thereby, represent a more equal background-ROI class ratio, the segmentation of abnormal medical features like lesions commonly reflects the strong class imbalance and can be characterized as more complex to evaluate. Furthermore, the imaging modality highly influences the ratio between ROI and background. Modern high-resolution imaging like whole-slide images in histopathology provides resolutions of 0.25 μm with commonly 80,000 × 60,000 pixels [19, 20] in which an anaplastic (poorly differentiated) cell region takes up only a minimalistic part of the image. In such a scenario, the resulting background-ROI class ratio could typically be around 18^3^:1 (estimated by a 512 × 512 ROI in an 80^3^ × 60^3^ slide). Another significant class ratio increase can be observed in 3D imaging from radiology and neurology. Computer tomography or magnetic resonance imaging scans regularly provide image resolutions of 512 × 512 pixels with hundreds of slices (z-axis) resulting in a typical class ratio around 373:1 (estimated by a 52 × 52 ROI in a 512 × 512x200 scan) [19]. In order to avoid such extreme imbalance bias, metrics that are distance-based like AHD or exclude true negative rewarding like DSC are recommended. Besides that, patching techniques (splitting the slide or scan into multiple smaller images) are often also applied to reduce complexity and class imbalance [2, 20].

### Influence of the segmentation task on evaluation

For valid interpretation of a MIS performance, it is crucial to understand metric behaviors and expected scores in different segmentation tasks. Depending on the ROI type like a lesion or organ segmentation, the complexity of the segmentation task and the resulting expected score varies significantly [21]. In organ segmentation, the ROI should be located consistently at the same position with low spatial variance between samples, whereas an ROI in lesion segmentation shows high spatial as well as morphological variance in its characteristics. Thereby, optimal performance metrics in organ segmentation are more likely to be possible, even though less realistic in lesion segmentation [22, 23]. This complexity variance implicates expected evaluation scores and should be factored in performance interpretation. Another important influencing factor in the segmentation task is the number of ROIs in an image. Multiple ROIs require additional attention for implementation and interpretation because not only high scoring metrics can be misleading and hiding undetected smaller ROIs between well predicted larger ROIs but also distance-based metrics are defined only on pairwise instance comparisons [21]. These risks should be considered in any evaluation of multiple ROIs.

### Multi-class evaluation

The previous evaluation metrics discussed are all defined for binary segmentation problems. It is needed to be aware that applying binary metrics to multi-class problems can result in highly biased results, especially in the presence of class imbalance [6]. This can often lead to a confirmation bias and promising-looking evaluation results in scientific publications which, however, are actually quite weak [6]. In order to evaluate multi-class tasks, it is required to compute and analyze the metrics individually for each class. Distinct evaluation for each class is in the majority of cases the most informative and comparable method. Nevertheless, it is often necessary to combine the individual class scores to a single value for improving clarity or for further utilization, for example as a loss function. This can be achieved by micro and macro averaging the individual class scores. Whereas macro-averaging computes the individual class metrics independently and just averages the results, micro-averaging aggregates the contributions of each class for computing the average score.

### Evaluation guideline

- Use DSC as main metric for validation and performance interpretation.
- Use AHD for interpretation of point position sensitivity (contour) if needed.
- Watch out for class imbalance and avoid interpretations based on high Accuracy.
- Provide next to DSC also IoU, Sensitivity, and Specificity for method comparability.
- Provide sample visualizations, comparing the annotated and predicted segmentation, for visual evaluation as well as to avoid statistical bias.
- Avoid cherry-picking high-scoring samples.
- Provide histograms or box plots showing the scoring distribution across the dataset.
- Keep in mind variable metric outcomes for different segmentation types.
- Be aware of interpretation risks by multiple ROIs.
- For multi-class problems, provide metric computations for each class individually.
- Avoid confirmation bias through macro-averaging classes which is pushing scores via background class inclusion.
- Provide access to evaluation scripts and results with journal data services or third-party services like GitHub [24] and Zenodo [25] for easier reproducibility.

#### Sample visualization

Besides the exact performance evaluation via metrics, it is strongly recommended to additionally visualize segmentation results. Comparing annotated and predicted segmentation allows robust performance estimation by eye. Sample visualization can be achieved via binary visualization of each class (black and white) or via utilizing transparent color application based on pixel classes on the original image. The strongest advantage of sample visualization is that statistical bias, overestimation of predictive power through unsuited or incorrect computed metrics, is avoided.

### Experiments

We conducted multiple experiments for supporting the principles of our evaluation guideline as well as demonstrate metric behaviors on various medical imaging modalities. Furthermore, the insights of this comment are based on the experience during the development and application of the popular framework MIScnn [18] as well as our contribution to currently running or already published clinical studies [2, 26–28].

The analysis utilized our medical image segmentation framework MIScnn [18] and was performed with the following parameters: Sampling in 64% training, 16% validation, and 20% testing sets; resizing into 512 × 512 pixel images; value intensity normalization via Z-score; extensive online image augmentation during training, common U-Net architecture [29] as neural network with focal Tversky loss function [30] and a batch size of 24 samples; advanced training features like dynamic learning rate, early stopping and model checkpoints. The training was performed for a maximum of 1000 epochs (68 up to 173 epochs after early stopping) and on 50 up to 75 randomly selected images per epoch. For metric computation and evaluation, we utilized our framework MISeval, which provides implementation and an open interface for all discussed evaluation metrics in a Python environment [31]. In order to cover a large spectrum of medical imaging with our experiments, we integrated datasets from various medical fields: Radiology–brain tumor detection in magnetic resonance imaging from Cheng et al. [32, 33], ultrasound–breast cancer detection in ultrasound images [34], microscopy–cell nuclei detection in histopathology from Caicedo et al. [35], endoscopy–endoscopic colonoscopy frames for polyp detection [36], fundus photography–vessel extraction in retinal images [37], dermoscopy–skin lesion segmentation for melanoma detection in dermoscopy images [38].

## Outlook

This work focused on defining metrics, their recommended usage and interpretation biases to establish a standardized medical image segmentation evaluation procedure. We hope that our guidelines will help improve evaluation quality, reproducibility, and comparability in future studies in the field of medical image segmentation. Furthermore, we noticed that there is no universal Python package for metric computations, which is why we are currently working on a package to compute metrics scores in a standardized way. In the future, we want to further contribute and expand our guidelines for reliable medical image segmentation evaluation.

## Data Availability

In order to ensure full reproducibility, the complete code of the analysis is available in the following public Git repository: https://github.com/frankkramer-lab/miseval.analysis. Furthermore, the trained models, evaluation results, and metadata are available in the following public Zenodo repository: https://doi.org/10.5281/zenodo.5877797. Our universal Python package for metric computation “MISeval: a metric library for Medical Image Segmentation Evaluation” is available in the following public Git repository: https://github.com/frankkramer-lab/miseval.

## Appendix

In the following chapters, each metric will be defined and discussed in terms of possible issues. Nearly all presented metrics, except Hausdorff distance, are based on the computation of a confusion matrix for a binary segmentation task, which contains the number of true positive (TP), false positive (FP), true negative (TN), and false negative (FN) predictions. Except for Cohen’s Kappa and Hausdorff distance, the value ranges of all presented metrics span from zero (worst) to one (best).

### F-measure based metrics

F-measure, also called F-score, based metrics are one of the most widespread scores for performance measuring in computer vision as well as in the MIS scientific field [10, 11, 39, 40]. It is calculated from the sensitivity and precision of a prediction, by which it scores the overlap between predicted segmentation and ground truth. Still, by including the precision, it also penalizes false positives, which is a common factor in highly class imbalanced datasets like MIS [10, 11]. Based on the F-measure, there are two popular utilized metrics in MIS: The Intersection-over-Union (IoU), also known as Jaccard index or Jaccard similarity coefficient, and the Dice similarity coefficient (DSC), also known as F1 score or Sørensen-Dice index. Besides that, the DSC is defined as the harmonic mean between sensitivity and precision, the difference between the two metrics is that the IoU penalizes under- and over-segmentation more than the DSC. Even so, both scores are appropriate metrics, the DSC is the most used metric in the large majority of scientific publications for MIS evaluation [10, 11, 40].

1 $$\mathrm{IoU}=\frac{TP}{TP+FP+FN}$$

2 $$\mathrm{DSC}=\frac{2TP}{2TP+FP+FN}$$

### Sensitivity and specificity

Especially in medicine, specificity and sensitivity are established standard metrics for performance evaluation [10, 11]. For pixel classification, the sensitivity (Sens), also known as recall or true positive rate, focuses on the true positive detection capabilities, whereas the specificity (Spec), also known as true negative rate, evaluates the capabilities for correctly identifying true negative classes (like the background class). In MIS evaluation, the sensitivity is a valid and popular metric, but still less sensitive to F-score based metrics for exact evaluation and comparison of methods [10, 11]. However, the specificity can result in an improper segmentation metric if not correctly understood. In MIS tasks, the specificity indicates the model’s capability to detect the background class in an image. Due to the large fraction of pixels annotated as background compared to the ROI, specificity ranges close to 1 are standard and expected. Thus, specificity is a suited metric for ensuring model functionality, but less for model performance.

3 $$\mathrm{Sensitivity}=\frac{TP}{TP+FN}$$

4 $$\mathrm{Specificity}=\frac{TN}{TN+FP}$$

### Accuracy/Rand index

Accuracy (Acc), also known as Rand index or pixel accuracy, is one or even the most known evaluation metric in statistics [10]. It is defined as the number of correct predictions, consisting of correct positive and negative predictions, compared to the total number of predictions. However, it is strongly discouraged to use accuracy due to the strong class imbalance in MIS. Because of the true negative inclusion, the accuracy metric will always result in an illegitimate high scoring. Even predicting the segmentation of an entire image as background class, accuracy scores are often higher than 90% or even close to 100%. Therefore, the misleading accuracy metric is not suited for MIS evaluation and using it is highly discouraged in scientific evaluations.

5 $$Accuracy=\frac{TP+TN}{TP+TN+FN+FP}$$

### Receiver operating characteristic

The ROC curve, short for Receiver Operating Characteristic, is a line plot of the diagnostic ability of a classifier by visualizing its performance with different discrimination thresholds [10]. The performance is shown through the true positive rate (TPR) against the false positive rate (FPR). In particular, ROC curves are widely established as a standard metric for comparing multiple classifiers and in the medical field for evaluating diagnostic tests as well as clinical trials [41]. As a single-value performance metric, the area under the ROC curve (AUC) was first introduced by Hanley and McNeil 1982 for diagnostic radiology [42]. Nowadays, the AUC metric is also a common method for the validation of machine learning classifiers. It has to be noted that an AUC value of 0.5 can be interpreted as a random classifier. The following AUC formula is defined as the area of the trapezoid according to David Powers [6]:

6 $$\mathrm{AUC}=1-\frac{1}{2}\left(\frac{FP}{FP+TN}+\frac{FN}{FN+TP}\right)$$

### Cohen’s kappa

The metric Cohen’s Kappa (Kap), introduced by Cohen in 1960 in the field of psychology, is a change-corrected measure of agreement between annotated and predicted classifications [10, 43, 44]. For interpretation, Kap measures the agreement caused by chance like the AUC score and ranges from -1 (worst) to + 1 (best), whereas a Kap of 0 indicates a random classifier. Through its capability of application on imbalanced datasets, it has gained popularity in the field of machine learning [44]. However, a recent study demonstrated that it still correlates strongly to higher values on balanced datasets [44, 45]. Additionally, it does not allow comparability on different sampled datasets or interpretation on prediction accuracy.

7 $${f}_{c}=\frac{\left(TN+FN\right)\left(TN+FP\right)+(FP+TP)(FN+TP)}{TP+TN+FN+FP}$$

8 $$Kap=\frac{\left(TP+TN\right)-{f}_{c}}{\left(TP+TN+FN+FP\right)-{f}_{c}}$$

### Average hausdorff distance

In contrast to other confusion matrix based metrics, the Hausdorff distance (HD) is a spatial distance based metric [10]. The HD measures the distance between two sets of points, like ground truth and predicted segmentation, and allows scoring localization similarity by focusing on boundary delineation (contour) [10, 46, 47]. Especially in more complex and granular segmentation tasks, exact contour prediction is highly important which is why HD based evaluations have become popular in the field of MIS [10]. However, because the HD is sensitive to outliers, the symmetric Average Hausdorff Distance (AHD) is utilized in the majority of applications instead [10, 17, 46]. The symmetric AHD is defined by the maximum between the directed average Hausdorff distance d(A,B) and its reverse direction d(B,A) in which A and B represent the ground truth and predicted segmentation, respectively, and ||a-b|| represents a distance function like Euclidean distance [10]:

9 $$\mathrm{d}(\mathrm{A},\mathrm{B})=\frac{1}{N}\sum_{a\in A}\underset{b\in B}{\mathrm{min}}|\left|a-b\right||$$

10 $$\mathrm{AHD}(\mathrm{A},\mathrm{B})=\mathrm{max}(d\left(A,B\right), d\left(B,A\right))$$

### Other metrics

In the field of MIS, various other metrics exist and can be applied depending on the research question and interpretation focus of the study. This work focused on the most suited metrics to establish a standardized MIS evaluation procedure and to increase reproducibility. For further insights on the theory of previously presented metrics or a large overview of all metrics for MIS, we refer to the excellent studies of Taha et al. [10]. Additionally, Nai et al. provided a high-quality demonstration of various metrics on a prostate MRI dataset [17].

## Abbreviations

MIS: Medical image segmentation
ROI: Region of interest
CDS: Clinical decision support
TP: True positive
FP: False positive
TN: True negative
FN: False negative
DSC: Dice similarity coefficient
IoU: Intersection-over-union
Sens: Sensitivity
Spec: Specificity
Acc: Accuracy
ROC: Receiver operating characteristic
TPR: True positive rate
FPR: False positive rate
Kap: Cohen’s kappa
HD: Hausdorff distance
AHD: Average hausdorff distance

## References

[1] Litjens G, Kooi T, Bejnordi BE, Setio AAA, Ciompi F, Ghafoorian M (2012). A survey on deep learning in medical image analysis. Med Image Anal.

[2] Müller D, Soto-Rey I, Kramer F (2021). Robust chest CT image segmentation of COVID-19 lung infection based on limited data. Inform Med Unlocked.

[3] Renard F, Guedria S, De Palma N, Vuillerme N (2020). Variability and reproducibility in deep learning for medical image segmentation. Sci Rep.

[4] Parikh RB, Teeple S, Navathe AS (2019). Addressing bias in artificial intelligence in health care. J Am Med.

[5] Zhang Y, Mehta S, Caspi A. Rethinking Semantic Segmentation evaluation for explainability and model selection. 2021. Accessed from: https://arxiv.org/abs/2101.08418

[6] Powers DMW. Evaluation: from precision, recall and F-measure to ROC, informedness, markedness and correlation. 2020. Accessed from: http://arxiv.org/abs/2010.16061

[7] El Naqa IM, Hu Q, Chen W, Li H, Fuhrman JD, Gorre N (2021). Lessons learned in transitioning to AI in the medical imaging of COVID-19. J Med Imaging.

[8] Gibson E, Hu Y, Huisman HJ, Barratt DC (2017). Designing image segmentation studies: statistical power, sample size and reference standard quality. Med Image Anal.

[9] Niessen WJ, Bouma CJ, Vincken KL, Viergever MA, Reinhard K, Siegfried HS, Max AV, Koen LV (2000). Error metrics for quantitative evaluation of medical image segmentation. Performance characterization in computer vision.

[10] Taha AA, Hanbury A (2015). Metrics for evaluating 3D medical image segmentation: analysis, selection, and tool. BMC Med Imaging.

[11] Popovic A, de la Fuente M, Engelhardt M, Radermacher K (2007). Statistical validation metric for accuracy assessment in medical image segmentation. Int J Comput Assist Radiol Surg..

[12] Sandeep Kumar E, Satya JP, Sujata D, Biswa RA, Mamta M, Ajith A, Arpad K (2020). Deep learning for clinical decision support systems: a review from the panorama of smart healthcare. Deep learning techniques for biomedical and health informatics.

[13] Altaf F, Islam SMS, Akhtar N, Janjua NK (2019). Going deep in medical image analysis: concepts, methods, challenges, and future directions. IEEE Access.

[14] Shaikh F, Dehmeshki J, Bisdas S, Roettger-Dupont D, Kubassova O, Aziz M (2021). Artificial intelligence-based clinical decision support systems using advanced medical imaging and radiomics. Curr Probl Diagn Radiol.

[15] Pedersen M, Verspoor K, Jenkinson M, Law M, Abbott DF, Jackson GD (2020). Artificial intelligence for clinical decision support in neurology. Brain Commun.

[16] Chen H, Sung JJY (2021). Potentials of AI in medical image analysis in gastroenterology and hepatology. J Gastroenterol Hepatol.

[17] Nai YH, Teo BW, Tan NL, O’Doherty S, Stephenson MC, Thian YL (2021). Comparison of metrics for the evaluation of medical segmentations using prostate MRI dataset. Comput Biol Med.

[18] Müller D, Kramer F (2021). MIScnn : a framework for medical image segmentation with convolutional neural networks and deep learning. BMC Med Imaging.

[19] Wolfgang Kuhlen T, Scholl I, Aach T, Deserno TM, Kuhlen T, Scholl I (2011). Challenges of medical image processing. Comput Sci Res Dev.

[20] Herrmann MD, Clunie DA, Fedorov A, Doyle SW, Pieper S, Klepeis V (2018). Implementing the DICOM standard for digital pathology. J Pathol Inform.

[21] Aydin OU, Taha AA, Hilbert A, Khalil AA, Galinovic I, Fiebach JB (2021). On the usage of average hausdorff distance for segmentation performance assessment: hidden error when used for ranking. Eur Radiol Exp.

[22] Isensee F, Jaeger PF, Kohl SAA, Petersen J, Maier-Hein KH (2021). nnU-Net: a self-configuring method for deep learning-based biomedical image segmentation. Nat Methods.

[23] Liu X, Song L, Liu S, Zhang Y, Feliu C, Burgos D (2021). Review of deep-learning-based medical image segmentation methods. Sustainability.

[24] GitHub. Accessed from: https://github.com/

[25] Zenodo—Research. Shared. Accessed from: https://zenodo.org/

[26] Müller D, Soto-Rey I, Kramer F. Multi-disease detection in retinal imaging based on ensembling heterogeneous deep learning models. In: studies in health technology and informatics. Accessed from: https://pubmed.ncbi.nlm.nih.gov/34545816/10.3233/SHTI210537 34545816

[27] Müller D, Soto-Rey I, Kramer F. An Analysis on ensemble learning optimized medical image classification with deep convolutional neural networks. 2022. Accessed from: http://arxiv.org/abs/2201.11440

[28] Meyer P, Müller D, Soto-Rey I, Kramer F, John M, Lăcrămioara ST, Catherine C, Arie H, Patrick W, Parisis G, Mihaela CV, Emmanouil Z, Oana SCh (2021). COVID-19 image segmentation based on deep learning and ensemble learning. Public health and informatics.

[29] Ronneberger O, Philipp Fischer, Brox T. U-Net: Convolutional Networks for Biomedical Image Segmentation. Lect Notes Comput Sci (including Subser Lect Notes Artif Intell Lect Notes Bioinformatics). 2015;9351:234–41.

[30] Abraham N, Khan NM. A novel focal tversky loss function with improved attention u-net for lesion segmentation. In: proceedings—international symposium on biomedical imaging. 2019.

[31] Müller D, Hartmann D, Meyer P, Auer F, Soto-Rey I, Kramer F, Sylvia P, Andrea P, Bastien R, Lucia S, Adrien U, Arriel B, Parisis G, Brigitte S, Patrick W, Ferdinand D, Cyril G, Jan DL (2022). MISeval: a metric library for medical image segmentation evaluation. Challenges of trustable AI and added-value on health. proceedings of MIE 2022.

[32] Cheng J, Yang W, Huang M, Huang W, Jiang J, Zhou Y (2016). Retrieval of brain tumors by adaptive spatial pooling and fisher vector representation. PLoS ONE.

[33] Cheng J, Huang W, Cao S, Yang R, Yang W, Yun Z (2015). Enhanced performance of brain tumor classification via tumor region augmentation and partition. PLoS ONE.

[34] Al-Dhabyani W, Gomaa M, Khaled H, Fahmy A. Dataset of breast ultrasound images. Data Br [Internet]. 2020 Feb 1 [cited 2022 May 12]; 28. Accessed from: https://pubmed.ncbi.nlm.nih.gov/31867417/10.1016/j.dib.2019.104863 PMC6906728 31867417

[35] Caicedo JC, Goodman A, Karhohs KW, Cimini BA, Ackerman J, Haghighi M (2019). Nucleus segmentation across imaging experiments: the 2018 data science bowl. Nat Methods.

[36] Bernal J, Sánchez FJ, Fernández-Esparrach G, Gil D, Rodríguez C, Vilariño F (2015). WM-DOVA maps for accurate polyp highlighting in colonoscopy: validation vs saliency maps from physicians. Comput Med Imaging Graph.

[37] Introduction—grand challenge. Accessed from: https://drive.grand-challenge.org/DRIVE/

[38] Codella NCF, Gutman D, Celebi ME, Helba B, Marchetti MA, Dusza SW, et al. Skin lesion analysis toward melanoma detection: a challenge at the 2017 International symposium on biomedical imaging (ISBI), hosted by the international skin imaging collaboration (ISIC). In: proceedings—international symposium on biomedical imaging. IEEE computer society; 2018. 168–72.

[39] Taghanaki SA, Abhishek K, Cohen JP, Cohen-Adad J, Hamarneh G (2021). Deep semantic segmentation of natural and medical images. Artif Intell Rev.

[40] Liu X, Song L, Liu S, Zhang Y (2021). A review of deep-learning-based medical image segmentation methods. Sustain.

[41] Kumar RV, Antony GM (2010). A Review of methods and applications of the ROC curve in clinical trials. Drug Inf J.

[42] Hanley JA, McNeil BJ (1982). The meaning and use of the area under a receiver operating characteristic (ROC) curve. Radiology.

[43] Cohen J (1960). A coefficient of agreement for nominal scales. Educ Psychol Meas.

[44] Cohen’s Kappa: what it is, when to use it, how to avoid pitfalls | KNIME. Accessed from: https://www.knime.com/blog/cohens-kappa-an-overview

[45] Delgado R, Tibau XA (2019). Why Cohen’s Kappa should be avoided as performance measure in classification. PLoS One.

[46] Aydin OU, Taha AA, Hilbert A, Khalil AA, Galinovic I, Fiebach JB (2021). On the usage of average hausdorff distance for segmentation performance assessment: hidden error when used for ranking. Eur Radiol Exp.

[47] Karimi D, Salcudean SE (2019). Reducing the hausdorff distance in medical image segmentation with convolutional neural networks. IEEE Trans Med Imaging.

